# Cancer Therapy Resistance: Choosing Kinase Inhibitors

**DOI:** 10.3390/pharmaceutics16030373

**Published:** 2024-03-07

**Authors:** Carmela Dell’Aversana, Federica Sarno, Rosaria Benedetti, Wouter Leonard Megchelenbrink, Donato Cappetta

**Affiliations:** 1 Institute of Experimental Endocrinology and Oncology “Gaetano Salvatore” (IEOS)-National Research Council (CNR), 80131 Napoli, Italy; 2 Department of Precision Medicine, University of Campania “Luigi Vanvitelli”, 80138 Naples, Italy; f.sarno@umcg.nl (F.S.); rosaria.benedetti@unicampania.it (R.B.); wouterleonard.megchelenbrink@unicampania.it (W.L.M.); 3 Department of Pathology and Medical Biology, University Medical Center Groningen, University of Groningen, 9713 GZ Groningen, The Netherlands; 4 Prinses Máxima Centrum for Pediatric Oncology, 3584 CS Utrecht, The Netherlands; 5 Division of Pharmacology, Department of Experimental Medicine, University of Campania “Luigi Vanvitelli”, 80138 Naples, Italy; donato.cappetta@unicampania.it

## 1. Introduction

Recent advances in comprehending the essential molecular mechanisms that govern cancer signaling have revealed the pivotal involvement of kinases in the development and progression of various cancer types. Additionally, kinases play a role in the acquisition of resistance to chemotherapeutics, targeted and endocrine treatments, and radiotherapy through the modulation of critical malignant molecular pathways [1,2]. Why are kinases crucial? By streamlining the oncological landscape, we find answers in the following: (1) the identification of over 500 enzymes capable of transferring a γ-phosphate group from ATP to serine, threonine, or tyrosine residues [3]; (2) the centrality of protein phosphorylation in cancer biology, influencing protein activity, folding, and interactions, thus determining the activation or deactivation of signaling pathways [4]; (3) uncovering genetically inherited variations in specific kinases through mutations across the entire genome, thus contributing to the initiation, promotion, progression, and recurrence of cancer [5]); and (4) the frequent overexpression of kinases with aberrant function in various cancer types [6]. It is not surprising, then, that phosphorylation represents a pharmacologically targetable mechanism, and there are numerous approved therapies for the treatment of cancer that focus on this process. It is crucial to recognize that the extensive array of protein kinase enzymes, characterized by a shared cofactor and a comparable three-dimensional structure of the catalytic site, coupled with the redundancy of activation signals and kinase-mediated signaling pathways, has posed challenges in the development of drugs with adequate selectivity. Nevertheless, the practical advantage of the polypharmacology exhibited by kinase inhibitors lies in their ability to serve in the treatment of various types of cancer with a single drug. Moreover, the capacity to inhibit multiple kinases, even with affinities varying significantly, within the same signal transduction pathway offers the prospect of attacking a cellular pathway from multiple angles, thereby optimizing the anticancer impact of the inhibitor [7].

The flipside associated with the redundancy of kinase signaling and its inhibition is that inhibitors, though theoretically effective against a tumor where that specific kinase is overexpressed or hyper-activated (gain of function), have often shown limited curative efficacy. Resistance to kinase inhibitors can be broadly categorized as inherent or primary resistance or acquired resistance. Even in cases where tumors carry an oncogenic driver mutation linked to heightened sensitivity to a specific kinase inhibitor, not all tumor cells exhibit a response, and others display only a transient period of benefit (e.g., cell heterogeneity as primary source of resistance). Acquired drug resistance is connected to the ability of cancer cells to sustain ongoing proliferative and survival signaling, despite the persistent presence of the original kinase inhibitor. Mechanisms of acquired resistance can be intrinsic, such as on-target secondary mutations, alternative ‘bypass’ signaling pathways, and histological transformation, or extrinsic, such as changes in TME and accessibility [8]. The strategies employed to enhance effectiveness have mainly been one of two types: (1) using combinations of multiple kinase inhibitors among themselves (drug combinations targeting single or parallel kinase pathways); and (2) combining kinase inhibitors with other anticancer regimens, especially those based on the immunological control of cancer proliferation [9]. The primary rationale for utilizing kinase inhibitors in combination with one another or with other interventions is to target the tumor directly (cytostatic effect) and, in parallel, inhibit the tumor’s immune-suppressive effects [10]. The objective is to shift the local microenvironment toward a proinflammatory state, thereby increasing the effectiveness of immune activators.

The targeting of kinases represents a new therapeutic paradigm in the clinical challenge of overcoming resistance. This Special Issue aims to provide an overview about promising findings and innovative therapeutic strategies to overcome cancer drug resistance, collecting both original research articles and topical reviews. This Special Issue adopts a strong focus on hot kinase-mediated molecular mechanisms driving resistance and novel therapeutic options with kinase inhibitor (KI) agents or combination therapies, including pharmaceutics strategies and technologies for drug optimization.

## 2. Overview of Published Articles

Machado et al. (contribution 1) discuss the application of KI in patients with relapsed/refractory (R/R) hematological malignancies. KI treatment regimens are clinically manageable, and outcomes are particularly effective when matched to tumor genetic profiles, giving rise to encouraging prospects of an era in which chemotherapy-free treatment regimens are a reality for many cancer patients. The use of KI as an alternative to traditional chemo-immunotherapy in oncology practice still faces obstacles in the non-specific activity of different first-generation inhibitors. However, in recent decades, investigations into their efficacy and the positive results achieved in clinical practice have given rise to encouraging prospects as new options for first- and second-line targeted therapies continue to emerge.

As analyzed by De Novellis et al. (contribution 2), the use of KIs as a preferable therapeutic approach, compared to conventional chemotherapy, is due to the greater selectivity toward neoplastic cells, higher efficacy rates, oral administration, and safer toxicity profile of KIs. Interestingly, studies in patients with CML have demonstrated that under predetermined conditions (CMR for at least 2 years after ≥3 years of treatment with TKI), the BCR-ABL1 inhibitor can be stopped and reactivated in case of molecular relapse. In contrast, TKI discontinuation is not yet recommended in CLL, as remissions are not common with TKI-based treatment (extremely rare MRD remissions), and a worse outcome has been described in patients who have undergone TKI discontinuation.

Despite the significant improvement and efficacy of targeted therapies based on TKIs in combination with immunological/pharmacological therapies for CML, several patients still develop an intolerance or resistance to TKIs. Amarante-Mendeset al. (contribution 3) underline that various resistance mechanisms are independent of the kinase or even BCR-ABL1, activating alternative survival pathways such as PI3K-AKT. Researchers are focused on developing alternative therapies that can collaborate constructively with existing treatments and improve the current approaches. Antioxidant therapies leading to reactive oxygen species inhibition, vascular growth factor receptor (VEGFR) inhibitors, or BH3 mimetic inhibitors (venetoclax) decrease TKI resistance [11,12]. Additionally, strategies aimed at degrading the BCR-ABL1 protein were tested using PROTAC (proteolysis-targeting chimera) technology, which demonstrated efficiently inhibited proliferation and induced apoptosis in vitro [13].

Recent advances in the development of small molecule KIs were also discussed by Zhong et al. (contribution 4) with regard to the novel treatment of resistant prostate cancer. Many of these designed inhibitors are in the preliminary stages of preclinical studies, and some are approved by the FDA. Among them are serine/threonine kinase inhibitors (IKK, TBK1, PKA, PIM kinase, Tpl2, GSK-3β, CDK, (PI3K)/AKT) and TKIs, such as lapatinib or dacomitinib (a dual inhibitor of EGFR and HER2, applied in a phase II), lipid kinases, and carbohydrate kinases (hexokinase and phosphofructokinase) [14]. However, treatment against a single kinase often is not enough. Double inhibition is required; for example, MET and VEGFR inhibition by a multi-target TKI, cabozantinib, showed much stronger anticancer activity than the inhibitors axitinib and crizotinib used alone [15]. Furthermore, the development of inhibitors/activators that could not only inhibit the abnormal activation of kinases in tumor cells to suppress proliferation but also modify the activity of kinases in immune/inflammatory cells to tilt the balance of the TME is under investigation.

Aggressive thyroid tumors also show frequent mutations in tyrosine kinase receptors in components of the MAPK/PI3K signalling pathway (RAS and BRAF) or in chromosomal rearrangements (RET/PTC and NTRK hybrids). Cuomo et al. (contribution 5) discuss TKI treatments as an effective therapeutic option for these aggressive tumors. Treatments for differentiated thyroid cancer include surgery and postsurgical thyroid ablation with radioiodine (RAI). However, TKIs can improve the prognosis of patients with advanced thyroid cancer as alternative treatment option for metastatic lesions, not responsive to surgery/RAI therapy.

The FDA and EMA have approved several TKIs as a treatment in refractory advanced or metastatic differentiated thyroid carcinomas. Sorafenib and lenvatinib, with progression-free survival rates of 10.8 and 18.3 months, respectively, are used as first-line treatments [16]. Cabozantinib is indicated as a second-line treatment [17]. However, the main limitations of TKIs are the significant adverse effects, limiting their use or dosage and leading to drug discontinuation or dose reduction. Additionally, the long-term administration of TKIs leads to the progressive inhibition of iodine uptake, acquired chemoresistance via the positive selection of dedifferentiated stem cells, and the induction of DNA mutations.

Kinase inhibitors are also used to treat triple-negative breast cancer (TNBC), in which the survival rate is worse than in other subtypes of breast cancer [18]. Several treatment options are available, such as chemotherapy, immunotherapy, radiation therapy, and surgery. However, TNBC is extremely difficult to treat due to the absence of ER, PR, and HER2. A combination of chemotherapy and surgery is typically used for TNBC patients, as it is a highly effective method of killing cancer cells throughout the body. Obidiro et al. (contribution 6) discuss the use of TKIs, in addition to conventional therapies, as a pharmacological approach for TNBC. The small molecule inhibitors, gefitinib and erlotinib, and the monoclonal antibody, cetuximab, are currently used as EGFR inhibitors in solid tumors. The combination of gefitinib, carboplatin, and docetaxel is a useful option in the treatment of TNBC. Treatment with bevacizumab, a monoclonal antibody that binds the VEGF receptor, is FDA approved as a treatment for TNBC. In ongoing studies, PI3K/AKT/mTOR and everolimus are still being studied for the treatment of TNBC [19].

Despite the great developments in TKIs, the side effects are so severe that they are only designated for progressive, debilitating, or life-threatening conditions. Moreover, low bioavailability and the mechanisms of resistance limit their use. These negative aspects can be addressed by designing next-generation TKIs or new drug formulae, in order to overcome pharmacological and pharmacokinetic difficulties. In this regard, Jampilek et al. summarized the results of the applications of self-nanoemulsifying drug delivery systems, nanoemulsions, liposomes, solid lipid nanoparticles (SLNPs), lipid-polymeric hybrid nanoparticles, and nanostructured lipid carriers (NLCs) used as TKI drug delivery systems in vitro and in vivo. The targeted delivery of kinase inhibitors using lipid-based delivery systems (liposomes, SLNPs, and NLCs) contributes to the reduction in side effects and improvement in drug efficiency in target organs, as demonstrated by the encapsulation of imatinib in nanosystems, which has proven to be an effective strategy to reduce the toxic effect caused by the drug itself [20].

In the same line of research, Bortnevskaya et al. (contribution 7) demonstrated the efficiency of the conjugation of photosensitizing therapy with low molecular weight TKIs and the use of micellar drug carriers as delivery vehicles. Specifically, the authors designed a new drug conjugate based on meso-arylporphyrin with the TKI erlotinib. The encapsulation of the new conjugated drug in Pluronic F127 nanomicelles improves its biological properties and EGFR inhibition. They showed that the conjugate drug was 1.8 times more toxic towards the EGFR-overexpressing cell line, and it significantly improved the life of erlotinib compared to its administration alone.

Alternately, El-Shenawy et al. (contribution 8) aimed to improve the pharmacokinetic characteristics of gefitinib (GFT), a TKI used as a first-line treatment for patients with advanced or metastatic non-small cell lung, colon, and breast cancer. Low solubility and low oral bioavailability limit its clinical application. In this study, the authors used cubosomal GFT nanoparticles (GFT-CNPs),known as one of the most important vesicular systems, to improve oral solubility and bioavailability. This study strongly suggests the possible use of GFT-CNPs as an oral vesicular system for the treatment of colon cancer. Studies have shown that the GFT-CNP system is very stable, providing a sustained GFT release rate in vitro and in vivo.

A major disadvantage of KIs is the pharmacological resistance mechanism after a long period of administration. Among various mechanisms, autophagy has recently been reported to promote chemoresistance in cancer cells by protecting against apoptosis and driving senescence. PKCη is a kinase involved in the cellular response to oxidative stress through its role in the regulation of autophagy and subsequent induction of senescence. Rotem-Dai et al., in their work (contribution 9), show that PKCη promotes autophagy induced by ER and oxidative stress and facilitates the transition from autophagy to senescence. The knockdown of PKCη reduces both autophagic flux and senescence markers. Furthermore, using autophagy inhibitors such as chloroquine and 3-methyladenine, it is shown that PKCη and autophagy are required, in response to oxidative stress, to establish senescence in breast cancer. Therefore, targeted therapy against PKCη could limit breast cancer cell survival and chemoresistance in response to drug-induced autophagy.

Finally, in contribution 10, Masłowska et al. designed and synthesized radio-conjugates of two known peptide-type inhibitors of the VEGF165/NRP-1 complex: A7R peptide and its shorter analogue, the branched peptidomimetic Lys (hArg)-Dab-Pro-Arg. The synthesized radio-conjugates were tested for their possible use as theragnostic-type radiopharmaceuticals for the imaging and therapy of tumors overexpressing Neuropilin-1 (NRP1). The overexpression NRP1 and its interaction with vascular endothelial growth factor-165 (VEGF165) is associated with tumor growth and metastasis. Therefore, compounds that block the interaction of VEGF165 with NRP-1 represent a promising strategy for imaging and treating NRP-1-related pathologies. Both peptide-type inhibitors have been coupled to a radionuclide chelator for diagnostic and therapeutic uses. However, the enzymatic degradation of both studied inhibitors caused an insufficient stability of the radio-compounds in human serum, indicating that further modifications are required to sufficiently stabilize the peptidomimetics.

## 3. Conclusions

TKIs are effective treatments for many types of cancer and represent one of the most significant medical discoveries of the last century. Their success is due to the countless mutations, dysregulation, and overexpression of protein kinases involved in a multitude of disease processes. Ongoing research attention has provided a comprehensive understanding of TKI biology to expand the clinical application of TKIs and overpower some of the major challenges, such as drug resistance, pharmacokinetic limitations, and side effects. TKIs can potentially be used as a treatment for the individual genetic profile of a patient’s tumor, contributing to the concept of personalized or precision medicine. Very promising research options are being investigated: (i) the application of TKIs in combination with other targeted therapies or conventional treatments (such as chemotherapy and immunotherapy) to improve the effectiveness of the treatment and overcome resistance mechanisms; (ii) the administration of TKIs before or after surgery to prevent recurrences or improve surgical results by reducing tumor sizes. Furthermore, the delivery of TKIs at tumor sites may be improved through mechanisms such as novel drug delivery systems, the application of nanoparticles, or targeted drug conjugates, which may further enhance drug delivery to tumor cells and minimize drug exposure to normal tissues. To accomplish this, it is crucial to devote attention to the development of a new generation of TKIs and innovative delivery systems, as well as promote more powerful and less invasive anti-cancer therapies.
